# Celiac Disease: A Review from Genetic to Treatment

**DOI:** 10.61186/ibj.4028

**Published:** 2023-12-02

**Authors:** Erfaneh Jafari, Niloufar Soleymani, Masoud Hamidi, Azar Rahi, Akram Rezaei, Reza Azizian

**Affiliations:** 1 Pediatric Infectious Disease Research Center, Tehran University of Medical Sciences, Tehran, Iran;; 2 Department of Food Hygiene, Islamic Azad University (Science and Research Branch), Tehran, Iran;; 3 École Polytechnique de Bruxelles-BioMatter Unit, Université Libre de Bruxelles (ULB), Brussels, Belgium;; 4 Department of Pathobiology, School of Public Health, Tehran University of Medical Sciences, Tehran, Iran;; 5 Department of Microbiology, School of Medicine, Tehran University of Medical Sciences, Tehran, Iran;; 6 Biomedical Innovation and Start-Up Association (Biomino), Tehran University of Medical Sciences, Tehran, Iran

**Keywords:** Celiac disease, Dipeptidyl peptidase 4, HLA-DQ antigens, Probiotics

## Abstract

Celiac disease is a complex disorder influenced by genetic and environmental factors. When people with a genetic predisposition to CD consume gluten, an inflammatory response is triggered in the small intestine, and this reaction can be alleviated by the elimination of gluten from the diet. The clinical manifestations of CD vary greatly from person to person and begin at a young age or in adulthood. Influence of genetic factors on CD development is evident in carriers of the DQ2 and/or DQ8 allele. HLA genotypes are associated with gut colonization by bacteria, particularly in individuals suffering from CD. In addition, beneficial gut microbes are crucial for the production of DPP-4, which plays a key role in immune function, as well as metabolic and intestinal health. Therefore, probiotics have been recommended as a complementary food supplement in CD.

## INTRODUCTION

Celiac disease is an autoimmune disorder that causes reactions of both the innate and adaptive immune system. CD is involved in the gastrointestinal tract infection, mainly the small gut. The persistent inflammatory reaction is triggered by consuming gluten-containing products (e.g., wheat, barley, and rye) and subsides when gluten is removed from the diet. The development of CD, with an estimated incidence of 1% in the Western population, is closely linked to the MHC class II molecules. Among CD patients, around 3% possesses HLA-DQ alleles. In addition, an increased risk of developing CD is observed in individuals whose first-degree relatives are affected by the disease. The role of genetic factors in CD development is evident in families in which at least one member has CD^[^^1^^-^^4^^]^.

**Indicators for CD**

The CD is an intricate disease triggered by various factors, including genetic and environmental elements. In people with a genetic predisposition (as an example, the presence of HLA genes such as HLA-DQ2 or HLA-DQ-8), exposure to gluten is necessary but not sufficient for disease development. The exposure of these individuals to gluten and environmental elements causes the activation of their innate and adaptive immune responses^[^^5^^]^. Therefore, the signs and symptoms of the disease can vary greatly from case to case. The clinical manifestation of CD ranges from asymptomatic (silent) to symptomatic (full-blown acute or chronic), associated with abdominal distension and pain, iron deficiency anemia, peripheral neuropathy, decreased bone mass, bone fractures, and elevated liver enzyme levels. Despite the frequency of diagnosis of chronic CD, there is a widespread belief that the boundaries of CD are blurred^[^^2^^,^^3^^]^.

**Diagnosis of CD**

CD is a long-term disease that affects the gut and often arises from an inability to absorb nutrients due to gluten intolerance^[^^2^^,^^6^^]^. In addition, the microbiota has a decisive role in developing autoimmune and atopic diseases^[^^7^^]^. CD can occur early in life or later in adulthood^[^^8^^]^ with varying symptoms, particularly typical gastrointestinal symptoms such as abdominal cramps, bloating, flatulence, diarrhea, vomiting, foul-smelling urine, and pale-colored stools (steatorrhea). However, some patients have alternative symptoms; i.e., non-intestinal manifestations or nutritional deficiencies. Common extraintestinal manifestations include late puberty, short stature, fatigue, and iron deficiency anemia^[^^9^^]^. CD can manifest itself very subtly, and it may be confused with IBS. Therefore, patients presenting with the above-mentioned signs and symptoms have to be screened for CD^[^^10^^]^. Serological testing of antibodies is important in the diagnosis of CD and depends on the ingestion of gluten. The positive tests of TTG-IgA/IgG and IgA-EMA, accompanied by a small intestine biopsy, will define the genetic tests for the evaluation of patients on a gluten-free diet^[^^11^^]^. In all patients with CD, DQ2 and/or DQ8 class II HLA types have been identified, although the major utility of a genetic test is its negative predictive value. Therefore, for ruling out CD, further testing is needed^[^^11^^,^^12^^]^. CD symptoms are characterized by intraepithelial lymphocytosis and small-bowel biopsies and are classified according to Marsh or Marsh-modified. If both serologic assessments and the biopsy are positive, CD diagnosis would be considered definite. However, if positive serologic assessments with excessive antibody titers accompany a negative biopsy, the adequate diagnosis of CD is halted^[^^13^^]^.

**Treatment of CD**

The beneficial options for CD treatment are categorized into the following strategies: (a) eliminating toxic gluten peptides before they reach the intestine, (b) regulating the immunostimulatory elements of toxic gluten peptides; (c) modifying intestinal penetrability; (d) modulating immune system and forming gluten tolerance; and (e) controlling the imbalance in the intestine microbiota via immunotherapy, a promising strategy for treating the IgE-mediated wheat allergy^[^^14^^]^. Currently, the gluten-free diet is not always the only treatment proposed for CD as it is ineffective in all patients^[^^13^^,^^15^^]^. Oral enzyme therapy is another attractive approach that inactivates gluten peptides in the digestive tract^[^^16^^]^. Some microorganisms contain proteases that can degrade gluten peptides rich in glutamine and proline residues^[^^17^^,^^18^^]^. Therefore, probiotic preparations are suggested as a complementary dietary treatment for CD patients. In adult patients with IBS, the consumption of predigested gliadins without α-gliadin peptides was found to improve disease indicators^[^^19^^,^^20^^]^.

**Genetic disposition of CD**

Genetic background has an important role in the predisposition to CD. The HLA-DQ2 haplotype (DQA1*0501-DQB1*0201) is present in the majority of CD-affected individuals (90%), while the HLA-DQ8 haplotype (DQA1*0301-DQB1*0302) is found in 5% of these cases, which carry at least one of the two DQ2 alleles, especially DQB1*0201^[^^21^^]^. Both haplotypes are involved in CD development and expressed on the surface of APCs. These cells feature a strong affinity for deamidated gluten-derived peptides, which bind and present to CD4 T cells in suburothelium, starting up the inflammatory cascade, which is a function of CD. Auto-antibodies against TG2, specifically anti-TG2 and anti- EMA, are responsible for the deamination of gluten in CD patients^[^^22^^,^^23^^]^. However, the most important alteration is intestinal damage, often characterized by villous atrophy, a scientific feature in most CD cases. The binding properties of gluten-derived peptides and the ability to elicit an immunologic reaction depend on the exact HLA-DQ molecules found in each individual, with a dose-based effect. HLA-DQ2.5 has the potential to bind to the most important variety of immune-dominant gluten peptides and also has the high ability to form strong complexes with these peptides on APC. Therefore, individuals with the HLA-DQ2.5 heterodimer have a higher risk of developing CD, especially if they carry two HLA-DQB1*02 alleles (double dose). Individuals with HLA-DQ8 or only the HLA-DQB1*02 allele (HLA-DQ2.2 receptor) have the lowest risk of developing CD, and those with only the HLADQA1*05 allele (HLA-DQ7.5 receptor) have the lowest likelihood for CD development^[^^22^^,^^24^^]^.

**Association of HLA-DQ with CD**

CD is a polygenic and multifactorial disease, with genetic and environmental factors involved in its pathogenesis. A strong association of HLA-DQ alleles with CD has been demonstrated in various studies^[^^25^^,^^26^^]^. The alleles HLA-DQA1*05 and HLA-DQB1*02 encode the HLA-DQ2 heterodimers α-subunit and β-subunit, respectively. These alleles can occur on identical chromosomes in *cis* configuration (DR3/DQ2 haplotype) or on homologous chromosomes, in *trans* configuration (DR5/DQ7 and DR5/DQ2 haplotypes). There are two types of DQ2 heterodimers: DQ2.5 (DQA1*0501/B1*0201) and DQ2.2 (DQA1*0201/B1*0202)^[^^27^^]^. Patients with DQ2.5 heterodimers have a higher risk for CD development than those with DQ2.2 heterodimers^[^^28^^]^. Although DQ2.2 molecules are structurally very similar to DQ2.5 molecules, the gluten peptide-binding properties of DQ2.2 are less pronounced. The number of HLA DQB1*0201 copies in CD patients may have important implications. Heterozygotes can synthesize 4 αβ-chain combinations, whereas in homozygotes, all HLA-DQ molecules are identical^[^^27^^,^^28^^]^. In determining the risk, the presence of the second β-chain appears to be critical, while that of the second α-chain is less significant^[^^28^^]^. Experimental dat a have shown a correlation between the number of HLA-DQ2.5 molecules and CD risk; HLA-DQ2.5 homozygotes can present gluten peptides more successfully on APCs than HLA-DQ2.5 heterozygotes, leading to five-fold CD risk^[^^27^^]^. The gene dose of the HLA-DQ2 alleles expressed by APC, determines the strength of the immune response. HLA-DQ2.5 homozygotes show maximal T-cell activation and pro-inflammatory response, while heterozygotes show much less pronounced responses. In immunological in vitro studies, having HLA-DQ2 homozygosity may change the progression of CD. This is because carrying DQB1*02 alleles leads to a significant influence on gene dosage, ultimately resulting in an increased likelihood of complications as seen in clinical settings.^[^^27^^,^^28^^]^.

**HLA type as a target for CD Treatment**

The most uncomplicated treatment for CD is a gluten-free diet, but major efforts have been taken to expand alternative remedies. Two different approaches pursued by researchers include blocking peptides and recombinant TCR ligands. Gluten in the intestine is markedly resistant to enzymatic digestion, leading to the formation of proteolytic-resistant gluten peptides by physiological processes. These peptides can effectively activate disease-associated T cells through an HLA-mediated technique. The goal of such therapies is to convert these naturally occurring T cell-stimulating substances into inhibitors of HLA-mediated antigen presentation. These therapies pay special attention to HLA-DQ2.5 due to its high prevalence in patients with CD and the thorough investigating immune-dominant epitopes associated with it^[^^29^^]^. Blocking peptides are short sections of deamidated gliadin that have been designed to attach to the HLA-DQ2 groove without activating gliadin-responsive T cells with reduced activation. Some peptides have demonstrated affinity for the HLA-DQ2 receptor and inhibited T-cell proliferation, potentially serving as HLA blockers. Recombinant TCR ligands are partial HLA molecules that encompass the α1 and β1 domains of the HLA-DQ molecule and are bound to unique antigenic peptides^[^^30^^]^. This method has shown promise in preclinical models when targeting for inactivation of gliadin-reactive T cells, although its in vivo efficacy and protection remain uncertain^[^^31^^]^. One challenge of this method is that the proposed strategies focused on HLA can also impact different immune responses. Moreover, the blockers have displayed moderate efficacy in inhibiting gluten-triggered T-cell activation in vitro ^[^^29^^,^^32^^]^.

**Microbiota and CD**

Factors contributing to CD include genetics, prenatal influences, infections, and microbiota composition^[^^33^^]^. Bacteria such as *Prevotella* spp. and *Streptococcus* spp. have been found more frequently in children under two years of age and adults suffering from CD ^[^^34^^,^^35^^]^. Toll-like receptors from pathogenic bacteria trigger the innate immune system, which activates pro-inflammatory cytokines and Th1, Th2, and Th17 responses^[^^35^^,^^36^^]^. Childhood infections can be considered a risk factor for CD^[^^37^^,^^38^^]^. A Swedish study found that childhood CD has the characteristics of an infectious disease^[^^39^^]^, which peaked between 1985 and 1996 and was also observed in 2001–2004^[^^40^^]^. Children born with CD showed increased bacterial populations such as *Clostridiales*, *Prevotella*, and *Actinomyces* in the jejunum^[^^41^^,^^42^^]^. About 48 species of *Prevotella *spp. have been isolated from humans, and the oral cavity is the most common site for isolating this bacterium. These bacteria have also been isolated from stool samples^[^^41^^,^^43^^,^^44^^]^.

**Microbiota and HLA in CD**

The development of gluten intolerance is associated with the stimulation of gluten-specific CD4^+^ T cells in the lamina propria and the increase in IL-15^[^^45^^]^. The presence of HLA-DQ2/8 haplotypes is directly related to CD. Studies have confirmed an increase in *Firmicutes* and *Proteobacteria* populations and a decrease in *Actinobacteria* and *Bifidobacteria* populations in infants with HLA-DQ2 and HLA-DQ8 haplotypes, which demonstrate an association between HLA genotypes and colonization of the intestine by bacteria that are typical in CD^[^^46^^]^. The HLA-DQ2/8 haplotype is also observed in a general population, suggesting that genetics alone does not explain the high prevalence of CD. Furthermore, the gut microbiota in infants with the HLA-DQ2/8 haplotype is influenced by the type of feeding, and breastfeeding has been shown to have a protective effect against CD^[^^47^^,^^48^^]^. 

**Fig. 1:**
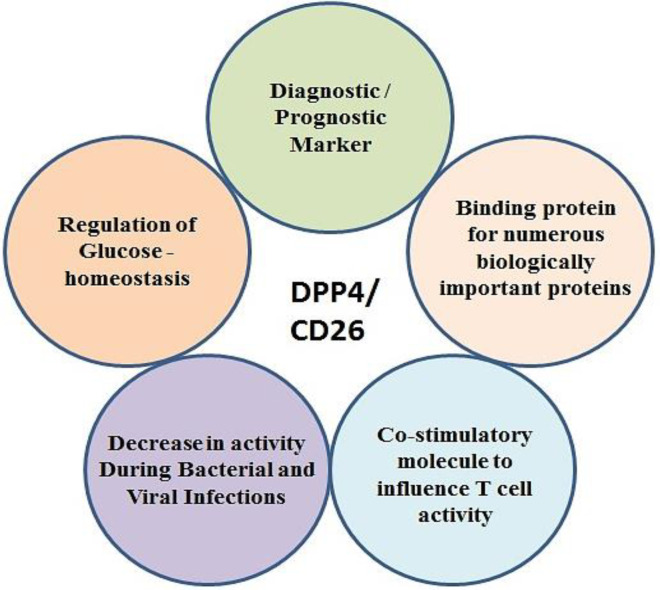
Roles attributed to DPP4

**DPP-4 and CD**

DPP-4 is an enzyme that influences metabolic behaviors and gut dysfunction by cleaving hormones and major peptides. This enzyme has various functions. It interacts with other proteins and is expressed in different tissues; therefore, DPP-4 could be used as a marker for a variety of diseases^[^^49^^,^^50^^]^ (Fig. 1). Beneficial microbes living in the intestine play an important role in the production of this enzyme. The presence of human DPP-4 homologs has been reported in some commensal bacteria such as *Lactobacillus* and *Prevotella *spp.^[^^51^^]^. Clinical studies have demonstrated that using prebiotic preparations such as barley, improves the growth of *Prevotella* spp. and positively affects the digestion and absorption of glucose^[^^52^^]^. Similarly, the activity of DPP-4 as Xaa-pro dipeptidyl-peptidase has been shown in lactic acid-producing bacteria such as *Lactobacillus*, *Lactococcus*, and *Streptococcus*^[^^53^^]^. Since DPP-4 has an active participation in the immune system, it can be considered a potential target for treating autoimmune diseases such as IBS^[^^50^^]^. Studies have exhibited a reduction in DPP-4 activity in Crohn's disease patients' bloodstream, plasma, and colon. However, the number of DPP-4-positive lymphocytes is higher in these patients than that of healthy ones^[^^54^^]^. A DPP-4-like homolog produced by intestinal commensals affects the digestion of dietary proteins; therefore, it can be a suitable host-side response to these foods^[^^51^^]^.

***Prevotella*** **spp. as a probiotic**

The main source of carbohydrate substrates available to the gut microbiota is the dietary fiber in the human diet. Therefore, it is possible to support the host's immune system by modifying the diet of the gut microbiota^[^^55^^]^. One of the most important mechanisms of probiotics in strengthening the host immune system is the prevention of colonization by occupying bacterial binding sites^[^^56^^]^. Researchers have exhibited that a diet rich in fiber, fat, and protein increases *Prevotella *spp. in the intestine, while a high consumption of fat and protein leads to an increase in *Bacteroides*^[^^57^^]^. Clinical studies have displayed that using prebiotic compounds such as barley as a dietary supplement, improves the growth of* Prevotella *spp. and positively affects the digestion and absorption of glucose^[^^58^^]^. Using barley as a prebiotic also results in a high *Prevotella/Bacteroides* ratio, which may benefit for cardiometabolic regulation. Therefore, the role of *Prevotella *spp. in improving the digestion and absorption of glucose has been confirmed^[^^59^^]^.

## CONCLUSION

CD is a multifaceted disorder that is affected by a combination of genetic and environmental factors. The clinical presentation of CD varies across individuals and can manifest at different stages of life influenced by the interplay between HLA haplotypes, feeding practices, and gut microbiota composition. Probiotics have emerged as a promising adjunct therapy for managing CD by promoting a healthy gut microbiome and optimizing immune function. Moreover, early identification and intervention in susceptible individuals, such as those with HLA haplotypes associated with increased CD risk, can aid in disease prevention. Moving forward, a complete approach that considers genetic susceptibility, microbiome modulation, and dietary interventions should be integrated into the management of CD to improve outcomes and enhance overall well-being.

## DECLARATIONS

### Acknowledgments

The proofreading of the present study was carried out with instatext (https://instatext.io). 

### Ethical approval

Not applicable.

### Consent to participate

Not applicable.

### Consent for publication

All authors reviewed and approved the final version of the manuscript.

### Authors’ contributions

EJ: formed the concept of the work, prepared the picture, and reviewed and edited the work; NS: wrote the initial draft; MH: reviewed and edited the work and prepared the work for submission; AzR and AkR: wrote the initial draft; RA: formed the concept of the work and prepared the work for submission. 

### Data availability

Not applicable. 

### Competing interests

The authors declare that they have no competing interests. 

### Funding

This research received no specific grant from any funding agency in the public, commercial, or not-for-profit sectors. 

### Supplementary information

The online version does not contain supplementary material.
